# Supplementary material for: The interactome of intact mitochondria by cross-linking mass spectrometry provides evidence for coexisting respiratory supercomplexes
Source: Mol Cell Proteomics. 2017 Dec 8;17(2):216–32. doi: 10.1074/mcp.RA117.000470 (PMC5795388; doi:10.1074/mcp.RA117.000470)
Supplement: Supplemental Data [file supp_RA117.000470_133922_0_supp_23978_fzffwf.zip › spectra_annotation/mito_spectra_annotation/366-1-4-1-6-1.pdf]

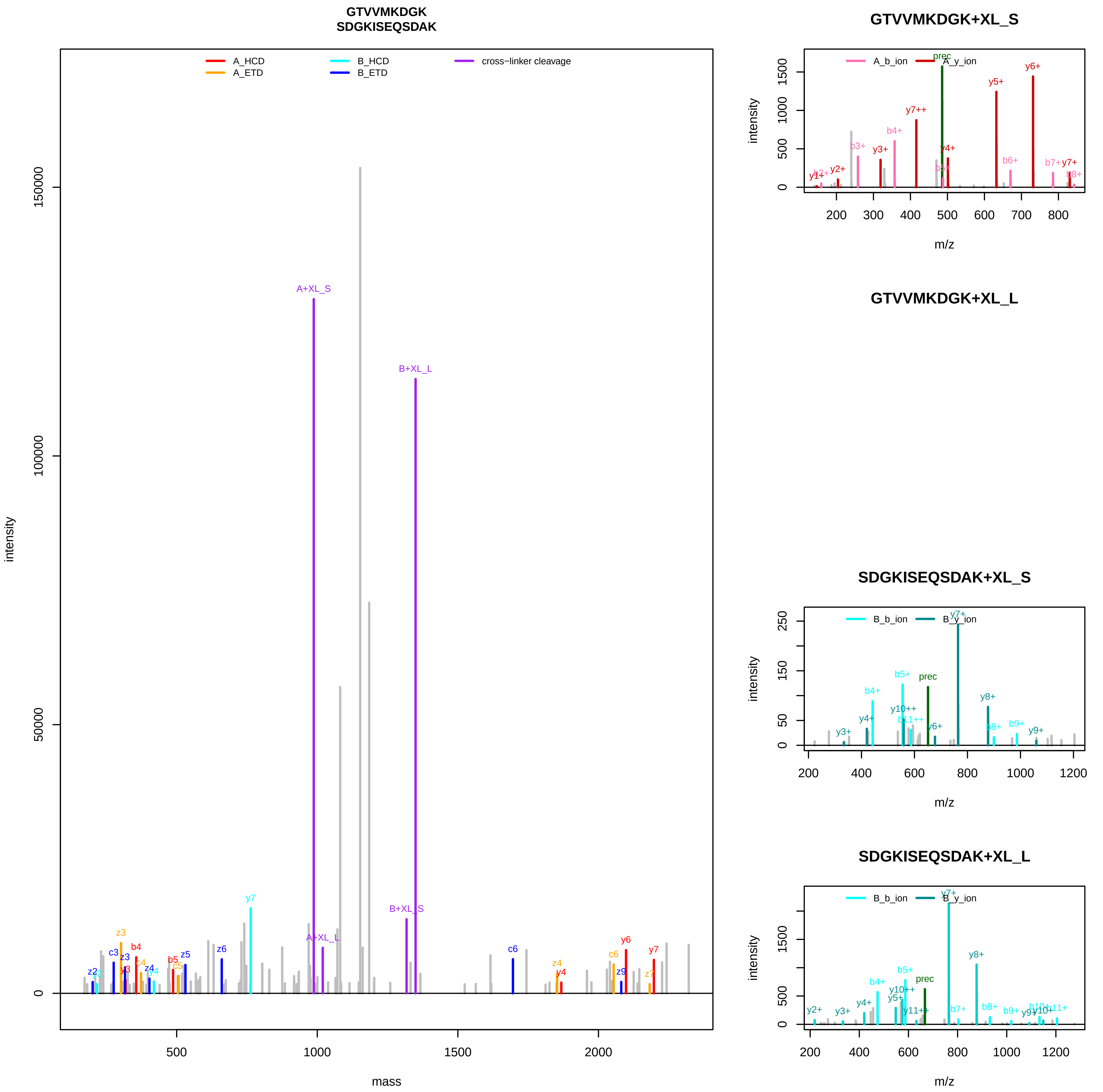

GTVVMKDGK
SDGKISEQSDAK
A_HCD
A_ETD
B_HCD
B_ETD
cross−linker cleavage
A+XL_S
B+XL_L
A+XL_L
B+XL_S
y7
z3
c3
b4
c4
b5
c5
z5
z6
c6
z4
y4
y6
z9
z7
intensity
mass
0
50000
100000
150000
500
1000
1500
2000
GTVVMKDGK+XL_S
A_b_ion
A_y_ion
prec
y6+
y5+
y7++
b4+
b3+
y3+
y4+
b6+
b7+
y7+
y2+
y1+
m/z
GTVVMKDGK+XL_L
SDGKISEQSDAK+XL_S
B_b_ion
B_y_ion
y7+
b5+
prec
b4+
y10++
y4+
y8+
b9+
y9+
y3+
y6+
SDGKISEQSDAK+XL_L
y8+
b5+
b4+
y10++
b7+
b8+
y2+
y3+
